# Investigation of Electrical Properties of the Al/SiO_2_/n^++^-Si Resistive Switching Structures by Means of Static, Admittance, and Impedance Spectroscopy Measurements

**DOI:** 10.3390/ma14206042

**Published:** 2021-10-13

**Authors:** Piotr Wiśniewski, Jakub Jasiński, Andrzej Mazurak, Bartłomiej Stonio, Bogdan Majkusiak

**Affiliations:** 1 Centre for Advanced Materials and Technologies CEZAMAT, Warsaw University of Technology, 02-822 Warsaw, Poland; Bartlomiej.Stonio@pw.edu.pl; 2 Center for Terahertz Research and Applications (CENTERA), Institute of High Pressure Physics, Polish Academy of Sciences, 01-142 Warsaw, Poland; 3 Institute of Microelectronics and Optoelectronics, Warsaw University of Technology, 00-662 Warsaw, Poland; jakub.jasinski@pw.edu.pl (J.J.); andrzej.mazurak@pw.edu.pl (A.M.); B.Majkusiak@imio.pw.edu.pl (B.M.)

**Keywords:** resistive switching, RRAM, memristor, silicon oxide, MIS structure, small-signal measurements

## Abstract

In this study, the resistive switching phenomenon in Al/SiO2/n^++^-Si structures is observed and studied by means of DC, small-signal admittance, and complex impedance spectroscopy measurements. Possible transport mechanisms in the high and low resistance states are identified. Based on the results of the applied measurement techniques, an electrical equivalent circuit of the structure is proposed. We discuss the effect of parasitic elements influencing the measurement results and show that a proper model can give useful information about the electrical properties of the device. A good agreement between the characteristics of the proposed equivalent circuit and the experimental data, based on different measurement procedures, confirms the validity of the used methodology and its applicability to the electrical characterization of RRAMs.

## 1. Introduction

The Resistive Random-Access Memory (RRAM) is a promising type of next-generation non-volatile computer memories due to its possible fast switching and low power consumption [1]. It offers promising properties for memory applications, hardware security, in-memory computing, and neuromorphic computing [2,3,4]. The latter application field, in particular, has attracted significant attention. It has been shown that RRAM devices can exhibit accumulative behavior whereby the resistance can be incrementally increased or decreased upon application of successive programming pulses of the same amplitude. This attribute can be used to emulate an artificial synapse, a crucial application for neuromorphic computing [5,6,7]. The two most studied types of RRAM devices are the conductive-bridge RAM (CBRAM), which is an electrochemical metallization memory (ECM) device, and the oxide RAM (OxRAM), a valence change memory (VCM) device [8]. The OxRAM is a device with an oxide resistive layer sandwiched between two inert electrodes. The inert electrode is an electrode that is not (or weakly) electrochemically active. Oxide ions and vacancies are generated upon application of the electric field across the layer. Vacancies form a conductive filament, which links electrodes as an electrical conduction path. A change in the polarization direction results in a disruption of the conductive filament due to Joule heating. Various transport processes in the oxide-based RRAM structures can be responsible for the resistive switching effect depending on the material stack and a fabrication method, e.g., trap-assisted tunneling, Poole-Frenkel emission, SILC, or hopping transport [9]. Many oxide materials exhibiting resistive switching (RS) have been examined in recent years, including HfO_2_, TiO_2_, and Ta_2_O_5_ [10,11,12,13,14,15,16,17,18,19]. Silicon oxide has also been tested as a potential candidate for an RS layer in CBRAM and OxRAM devices [20,21,22,23,24,25,26,27]. It would be a promising candidate for these applications due to its well-known properties and fabrication techniques [28,29]. However, there is only a limited number of works related to SiO_2_ as an RS layer, and further studies are needed.

Many works on the study of memristor devices concern the metal–insulator–metal (MIM) configuration. The metal–insulator–semiconductor (MIS) structure may be favorable in terms of compatibility with standard CMOS technology. Usually, a memory cell comprises an RRAM device and a selector device, which can be an MOS field-effect transistor. In this case, we have a 1T-1R memory cell configuration. The MIS structure can be easily incorporated into the transistor architecture as a part of the drain region, and the combined device consists of a resistive storage node in series with a select transistor. This type of device is called Contact RRAM (CRRAM) [30,31,32]. This work shows that the MIS silicon diode can also exhibit resistive switching properties. Similar structures were analyzed in the past but in different material configurations [22,23,33,34,35,36,37,38]. Most of the works regarding RRAM focus on DC measurement-based electrical characterization and extracting a possible conduction mechanism of the switching layer. We show and analyze the measurement results obtained for Al/SiO_2_/very highly doped Si(n) structures with the use of the admittance and impedance spectroscopy measurements. We show that a change in the compliance current results in different conductance levels, which is common for some types of RRAM devices [39,40]. Possible transport mechanisms in the high resistance state (HRS) and the low resistance state (LRS) are indicated and identified. An equivalent circuit of the structure is proposed, which can provide useful information about the RS layer. The results of the work give new insight into the possible origins of the RS effect in the investigated structures. 

## 2. Materials and Methods

**Figure 1:**
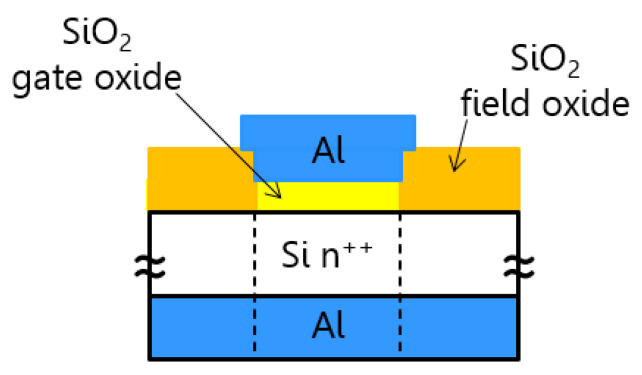
Cross-view of the investigated Al/SiO_2_/n^++^-Si device (not to scale).

MIS structures were fabricated using standard CMOS compatible processes: wet processing, photolithography, thermal oxidation, and magnetron sputtering. We used 2” n-type highly doped (arsenic) wafers with the resistivity in the range 0.001–0.005 Ω∙cm from Siegert Wafer Gmbh (Aachen, Germany). Wafers were cleaned using the standard RCA method. The field oxide was fabricated in a high-temperature furnace using a wet oxidation process. Then, the windows were opened using photolithography and wet etching with HF acid. Subsequently, a thin silicon oxide layer (5–6 nm) was grown in a dry thermal oxidation process (10 min at 820 °C). Aluminum gate electrodes were made using the lift-off process after the photolithography and metal deposition processes. Then, the bottom oxide layer was etched, and bottom Al metallization was formed. Structures were annealed in H_2_/Ar atmosphere in 400 °C for 30 min. The schematic cross-section of the investigated devices is presented in Figure 1.

**Figure 2:**
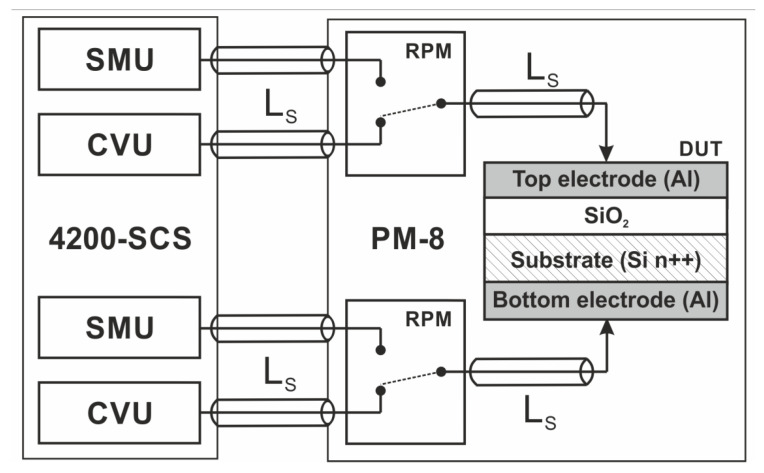
Measurement setup used to characterize fabricated structures.

Electrical measurements were made using Keithley 4200-SCS Semiconductor Characterization System (Keithley Instruments, LLC, Solon, OH, USA) combined with Süss MicroTec PM8 low noise probe shield. DC current–voltage characteristics were measured with the static source-measure unit (SMU), whereas the admittance and impedance measurements were carried out using the small-signal capacitance–voltage unit (CVU). Both units were connected to the device under test (DUT) through the ultra-fast remote switching module (RPM). All measurements were carried out at room temperature. The measurement setup is presented in Figure 2.

## 3. Results and Discussion

### 3.1. DC Measurements

**Figure 3:**
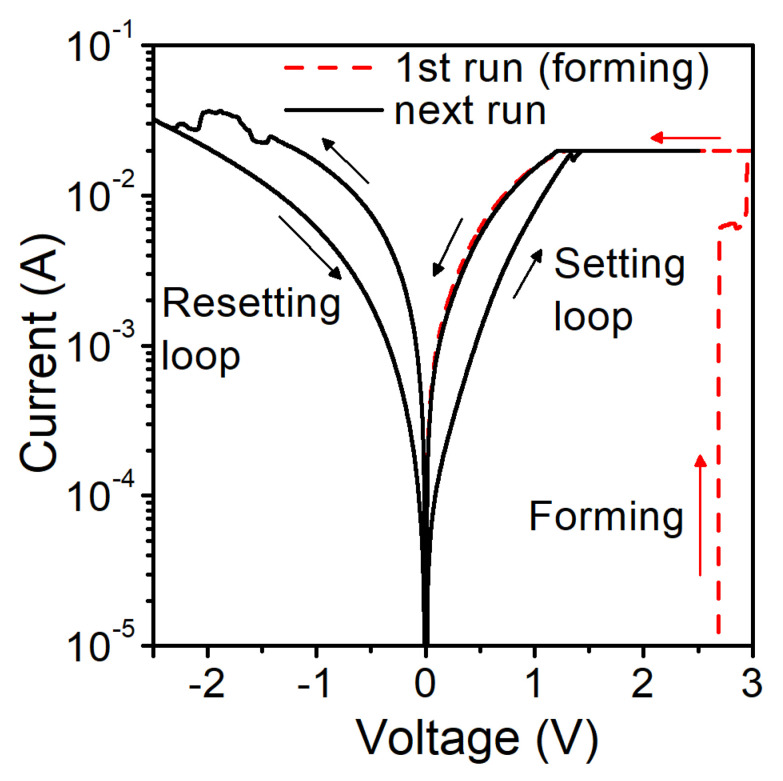
Measured current-voltage characteristics of Al/SiO2/n^++^ Si RRAM structure S1 with a gate diameter of 156 μm and CC = 20 mA.

**Figure 4:**
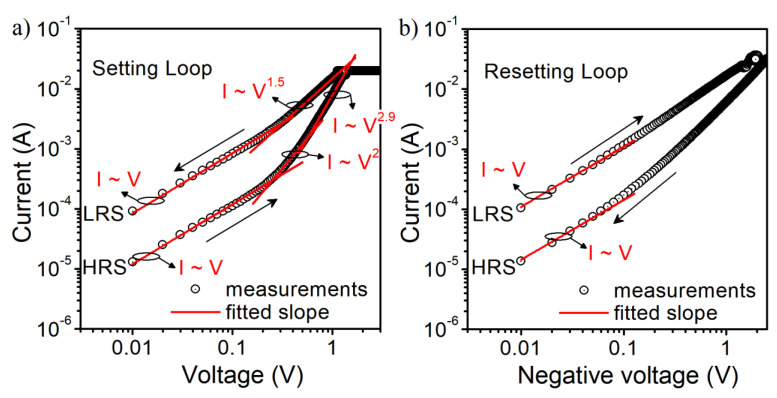
Current-voltage characteristics of Al/SiO_2_/n^++^ Si RRAM structure S1 with the gate diameter of 156 μm and CC = 20 mA at SET (**a**) and RESET (**b**) cycle with fitted curves of different slopes.

In Figure 3, we show the current–voltage characteristics of an Al/SiO_2_/n^++^ Si structure with a gate pad diameter of 156 μm (S1). Initial electroforming voltage is above 2.5 V. Structures were measured with the compliance current (CC) of 20 mA. For a given CC, the set voltage is above 1.2 V. We identify the transport mechanism as the space charge limited current (SCLC). In Figure 4, we show the slope of I–V curves at different states and voltage ranges. In the high-resistance state (HRS) of the SET cycle, we observe that initially, the current is proportional to the applied voltage (Ohmic conduction), and then it obeys Child’s quadratic law, which is related to partially filled traps [41,42]. In the high field region, a higher slope of the curve is observed, which is related to fully filled traps. In the low-resistance state (LRS), we have mainly Ohmic conduction. In the RESET cycle, the Ohmic conduction is mainly observed at low voltages. At higher voltage values, carrier transport through the dielectric is a mix of different types of transport mechanisms, and it is hard to identify it in a simple way.

**Figure 5:**
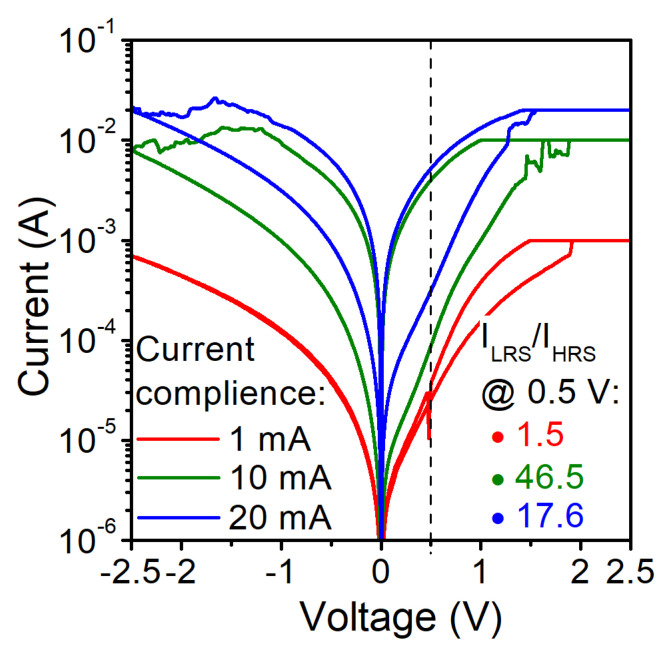
Measured current-voltage characteristics of Al/SiO2/n^++^ Si RRAM structure S2 with the gate diameter of 74 μm and various compliance set currents.

Figure 5 shows the current–voltage characteristics of the structure S2 with the gate diameter of 74 μm for different CC values. The initial forming voltage is above 2.0 V. At CC = 1 mA, we have not observed a reset current–voltage loop. For a higher CC, we observe an increase in the current in both the high resistance state (HRS) and the low resistance state (LRS). The CC level can be used to modify the conductance of the RRAM structure. This is probably due to the fact that a higher CC increases the size or number of conductive filaments (CF) within the structure [43]. For small CC values, this effect is less pronounced. CFs are probably not completely formed, unstable and non-persistent, and thus, they can be easily dissolved using a very small voltage value.

### 3.2. Small-Signal Measurements

**Figure 6:**
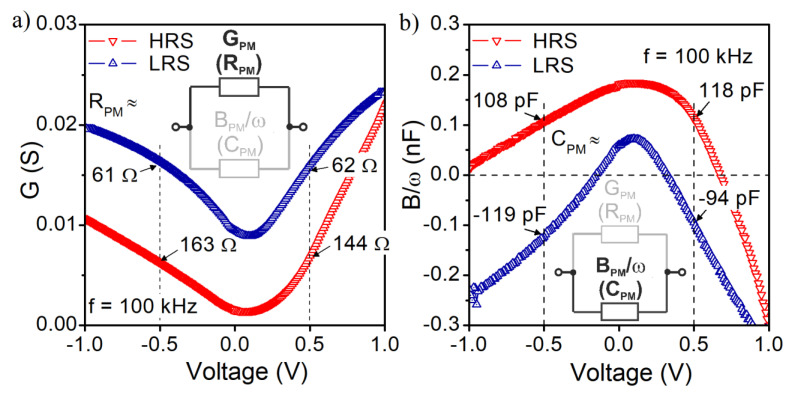
Small-signal admittance components for S1 structure at the frequency f = 100 kHz in different resistance states versus the gate bias voltage. (**a**) conductance; (**b**) susceptance.

In our study, we also used the small-signal measurement technique to obtain the admittance characteristics of the device. In Figure 6, we show conductance and susceptance of S1 structure measured at the frequency f = 100 kHz. Measurements were carried out in a limited gate voltage range to maintain the structure in one of the states, HRS or LRS.

**Figure 7:**
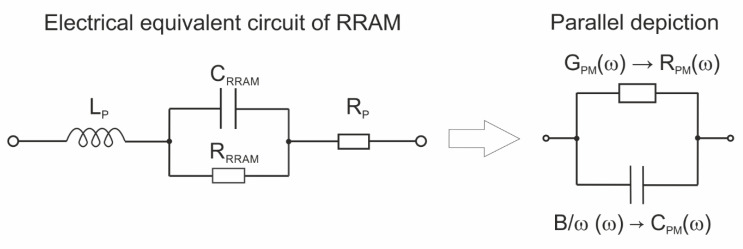
Electrical equivalent circuit of RRAM device with series parasitic components and its measurement parallel depiction.

As expected, the conductance in LRS is higher than in HRS (Figure 6a). The measurement data taken in the parallel equivalent circuit were used to extract R_PM_ values at −0.5 V and +0.5 V, as marked in Figure 6a. The imaginary part of the measured admittance is presented in Figure 6b. In the HRS state, the susceptance is positive for negative gate voltages up to −1.0 V, indicating the capacitive behavior of the structure. While moving towards positive gate voltages, one can observe that susceptance becomes negative in the bias range above 0.65 V. The closer to the set voltage, the lower susceptance is observed, and the characteristic tends to the curve representing the susceptance in the LRS. In the LRS, the structure behavior is mainly inductive within the considered gate voltage range. Only in a limited voltage range close to 0.0 V, the structure exhibits capacitive behavior. In general, the admittance of a considered device can be described by a simple electrical equivalent circuit, which is presented in Figure 7.

It is particularly important that the series parasitic inductance (L_P_) and resistance (R_P_) cannot be omitted when the resistance (R_RRAM_ in Figure 7) in the parallel RC circuit of RRAM structure becomes low, and the capacitance C_RRAM_ simultaneously becomes high. Such a situation occurs when a conductive filament inside RRAM dielectric is formed. As a result, the admittance of the electrical equivalent circuit presented in Figure 7 can be calculated as follows:

(1) $$Y={\left(R_{P}+j\omega L_{P}+\frac{1}{1/R_{RRAM}+j\omega C_{RRAM}}\right)}^{-1}$$

The conductance and susceptance of the measurement parallel depiction of the considered equivalent circuit are described by the following formulae:

(2) $$G=\frac{R_{P}+R_{RRAM}{\left(1+A\right)}^{-1}}{{\left[R_{P}+R_{RRAM}{\left(1+A\right)}^{-1}\right]}^{2}+\omega^{2}{\left[L_{P}-{\left(\omega^{2}C_{RRAM}\left(1+A^{-1}\right)\right)}^{-1}\right]}^{2}}$$

(3) $$B=\omega \frac{{\left(\omega^{2}C_{RRAM}\left(1+A^{-1}\right)\right)}^{-1}-L_{P}}{{\left[R_{P}+R_{RRAM}{\left(1+A\right)}^{-1}\right]}^{2}+\omega^{2}{\left[L_{P}-{\left(\omega^{2}C_{RRAM}\left(1+A^{-1}\right)\right)}^{-1}\right]}^{2}}$$

where *A* is equal to ${\left(\omega C_{RRAM}R_{RRAM}\right)}^{2}$.

The sign of the susceptance is determined by the sign of the numerator. In LRS, the capacitance of the RRAM device (C_RRAM_) increases according to the increase in the absolute value of the applied voltage. It results from a shortening gap between the growing filament and the top electrode of the device. In such a situation, the parasitic inductance (L_P_) can dominate the numerator of Equation (3), imposing the negative value of the measured susceptance. A similar mechanism can be a reason for the negative susceptance of the investigated structures in LRS (Figure 6b).

### 3.3. Complex Impedance Spectroscopy

Some information on the electrical properties of a resistive switching layer (e.g., filaments) and a RRAM device can be extracted from the impedance spectroscopy [44,45,46]. In many cases, a simple parallel RC equivalent circuit is quite enough to approximate the conducting behavior of the investigated structure/dielectric layer in a wide range of frequencies. However, in some situations, it is necessary to extend a simple equivalent RC network by additional parallel RC circuits. For example, it may be dictated by a different behavior of the RRAM structure in LRS and HRS modes.

**Figure 8:**
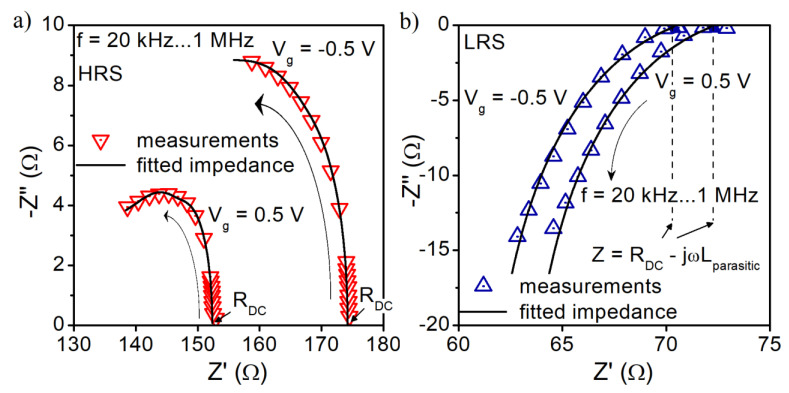
Complex impedance spectra of investigated structure (S1) for a frequency range of 20 kHz–1 MHz at different gate bias voltage values in (**a**) HRS and (**b**) LRS.

**Figure 9:**
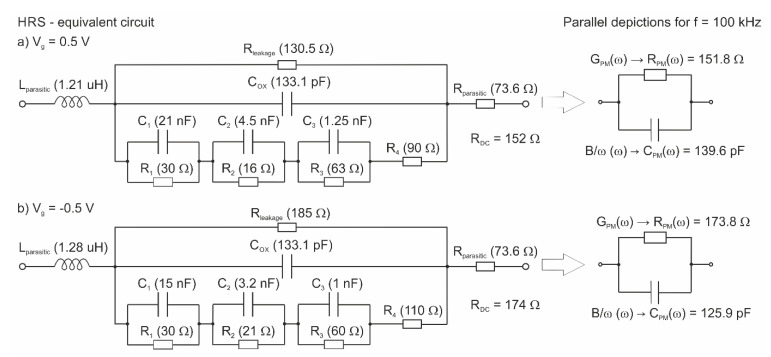
Electrical equivalent circuit for the measured device in HRS at two bias points, (**a**) V_g_ = 0.5 V (**b**) V_g_ = −0.5 V.

**Figure 10:**
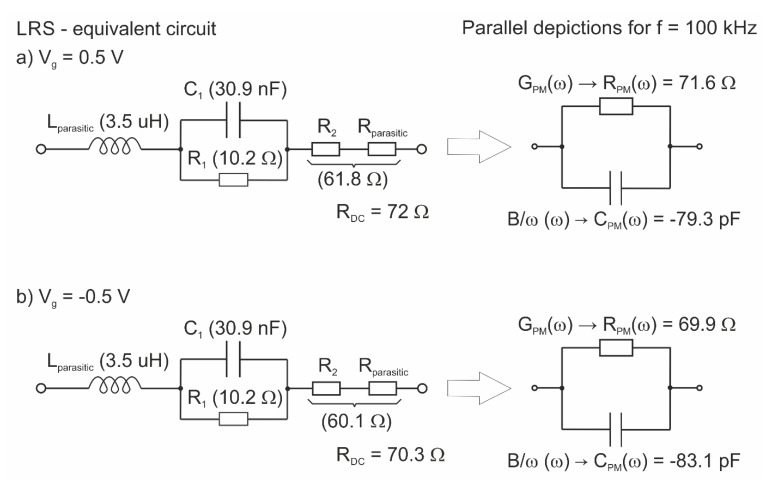
Electrical equivalent circuit for the measured device in LRS at two bias points, (**a**) V_g_ = 0.5 V; (**b**) V_g_ = −0.5 V.

Figure 8a,b present the complex impedance (Z″-Z′) of the investigated structure at a given bias voltage (+0.5 V and −0.5 V) for HRS and LRS. The figures combine the measured and simulated data for the electrical equivalent circuits presented in Figure 9 and Figure 10. In our considerations, we assume that a highly doped substrate of the device behaves like a metal electrode. Thus, the elements of the proposed models correspond to the switching layer of the RRAM device and to the parasitic parameters of the measurement setup. A very good match of the measured and simulated data for both HRS and LRS in the frequency range of 20 kHz–1 MHz was obtained.

In Figure 9, we present the electrical equivalent circuit for the measured device in HRS at two bias points. We have three parallel branches representing the electrical behavior of the insulator layer. The capacitance C_ox_ represents the capacitance related to the gate insulator of the device (its value directly corresponds to the approximately 5 nm thick SiO_2_ layer and the gate electrode diameter of 156 um). Leakage resistance (R_leakage_) represents the gate leakage current that results from tunneling or other transport mechanisms, different than the transport through conducting filaments. The third branch, comprising series resistance and parallel RC circuits, represents the cumulative electrical behavior of conductive filaments. R_parasitic_ describes the spread parasitic resistance brought in by the measurement setup and the series resistance of the device. L_parasitic_ represents the uncompensated inductance of the wiring and the switching matrix in the measurement setup (Figure 2). 

The shape of the fitted impedance at HRS (Figure 8) is mainly determined by a combination of complex semi-circles related to the corresponding parallel RC network. The remaining elements of the proposed equivalent circuit are responsible for the position of the curve at the Z-plane and have less impact on its shape. The branch containing the series connection of the parallel RC circuits and the resistance R_4_ reflects the electrical behavior of the conducting filament(s).

In the case of LRS, the proposed equivalent circuit of the investigated structure is as presented in Figure 10. The conducting behavior of the switching layer is represented only by a single RC network. Moreover, the equivalent circuit consists series of parasitic elements (R_parasitic_, L_parasitic_). During switching on, the filament increases its volume, so the resultant value of spread resistance of the device decreases. At the same time, the gap between the filament and the top electrode decreases substantially. It is represented by capacitance, the value of which is greater than the value of capacitances corresponding to filament structure at HRS. 

The parallel RC network (Figure 10) is mainly responsible for the shape of the fitted impedance at LRS. The series connection of L and R is represented by a straight line on Z-plane (dashed lines in Figure 8b). As in the proposed equivalent circuit, adding parallel capacitance is necessary to match the simulated data to the measured ones. C_OX_ and R_leakage_ were omitted in the electrical equivalent circuit in LRS because their values are negligible compared to C and R in a parallel RC network (Figure 10). 

**Figure 11:**
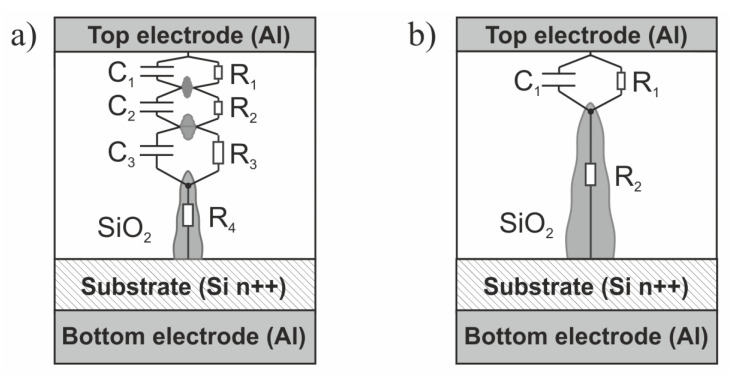
The sketch of the RS structure and its electrical model in HRS (**a**) and LRS (**b**).

In Figure 11, we propose a sketch of a hypothetical structure of a conducting filament; the electrical behavior is approximated by proposed equivalent circuits, with the interpretation of different R and C elements in the structure in HRS and LRS.

A negative value of the susceptance at LRS (Figure 6b) and a negative imaginary component of Z (Figure 8b) is often regarded as proof of an inductive behavior of the structure/conducting filaments [25,44,47]. However, such a behavior can be caused by parasitic uncompensated inductance (L_S_ in Figure 2) of the measurement setup (wiring, switching matrix, etc.). In our considerations, a suitable agreement between the simulated and measured data was obtained for the parasitic inductance; its value of 3.5 μH is comparable to L_S_. This gives rise to the claim that the L_parasitic_ value in our model is closely related to the measurement setup, rather than the conduction mechanism within the RRAM structure. Based on the obtained measurement, we believe that the studied structure is an OxRAM device. However, further studies are needed to confirm this claim.

**Figure 12:**
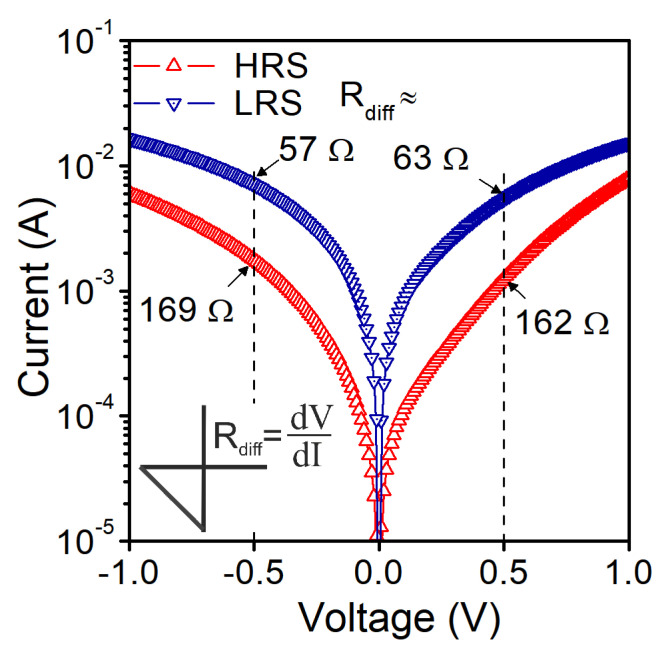
R_diff_ values extracted from the measurements of S1 structure at different resistance states and considered gate bias voltages.

The parallel depictions of the proposed equivalent circuits (at HRS and LRS for f = 100 kHz) consist of elements (G_PM_, C_PM_) whose values correspond to the measured ones marked in Figure 6a,b—real and imaginary parts of admittance, respectively. Static equivalent resistance (R_DC_) value extracted from the proposed equivalent circuits agrees with the differential resistance R_diff_ values obtained from the measured static I–V characteristics for both considered bias voltages at HRS and LRS (Figure 12). 

## 4. Conclusions

In this work, current–voltage characteristics of the experimental Al/SiO_2_/n^++^ Si RRAM structures are presented and analyzed. An influence of the compliance current is shown. The small-signal admittance and complex impedance measurements are used to characterize the structure and propose its electrical equivalent circuit. A suitable agreement between characteristics of the proposed equivalent circuit and the experimental data based on different measurement procedures (DC, small-signal admittance, complex impedance spectroscopy) confirms that the used methodology can be a useful technique for investigating electrical properties of RRAM devices, giving new insights into the origins of the resistive switching phenomenon.

## Data Availability

The data presented in this study are available on reasonable request from the corresponding author.
